# The impact of internet use on residents’ happiness in China

**DOI:** 10.3389/fpubh.2023.1188259

**Published:** 2023-08-24

**Authors:** Yongqiang Sun, Jing Gao, Xiaolin Zhang, Yaoxing Cheng

**Affiliations:** School of Economics, Minzu University of China, Beijing, China

**Keywords:** internet use, social interaction frequency, socioeconomic status, happiness, intermediary effect

## Abstract

Using data from the 2018 Chinese General Social Survey (CGSS), this study investigates the impact of internet use on residents’ happiness. Empirical results show that internet use significantly enhances residents’ happiness. Considering the possible endogeneity problem, a two-stage tool variable estimation is carried out with ownership of a mobile phone as the tool variable. After overcoming the endogenous problem and conducting a series of robustness tests, the conclusion is still valid. The action mechanism finds that social interaction frequency and socioeconomic status (SES) play significant mediating effects in the process of internet use affecting happiness. Specifically, internet use significantly increases the social interaction frequency of residents and enhances their SES. The improvement of social interaction frequency and SES will significantly increase residents’ happiness. Therefore, this paper gives policy recommendations from the perspectives of regulating and deepening internet use, increasing the frequency of communication among residents, and improving their SES to better enhance their happiness.

## 1. Introduction

**Figure 1:**
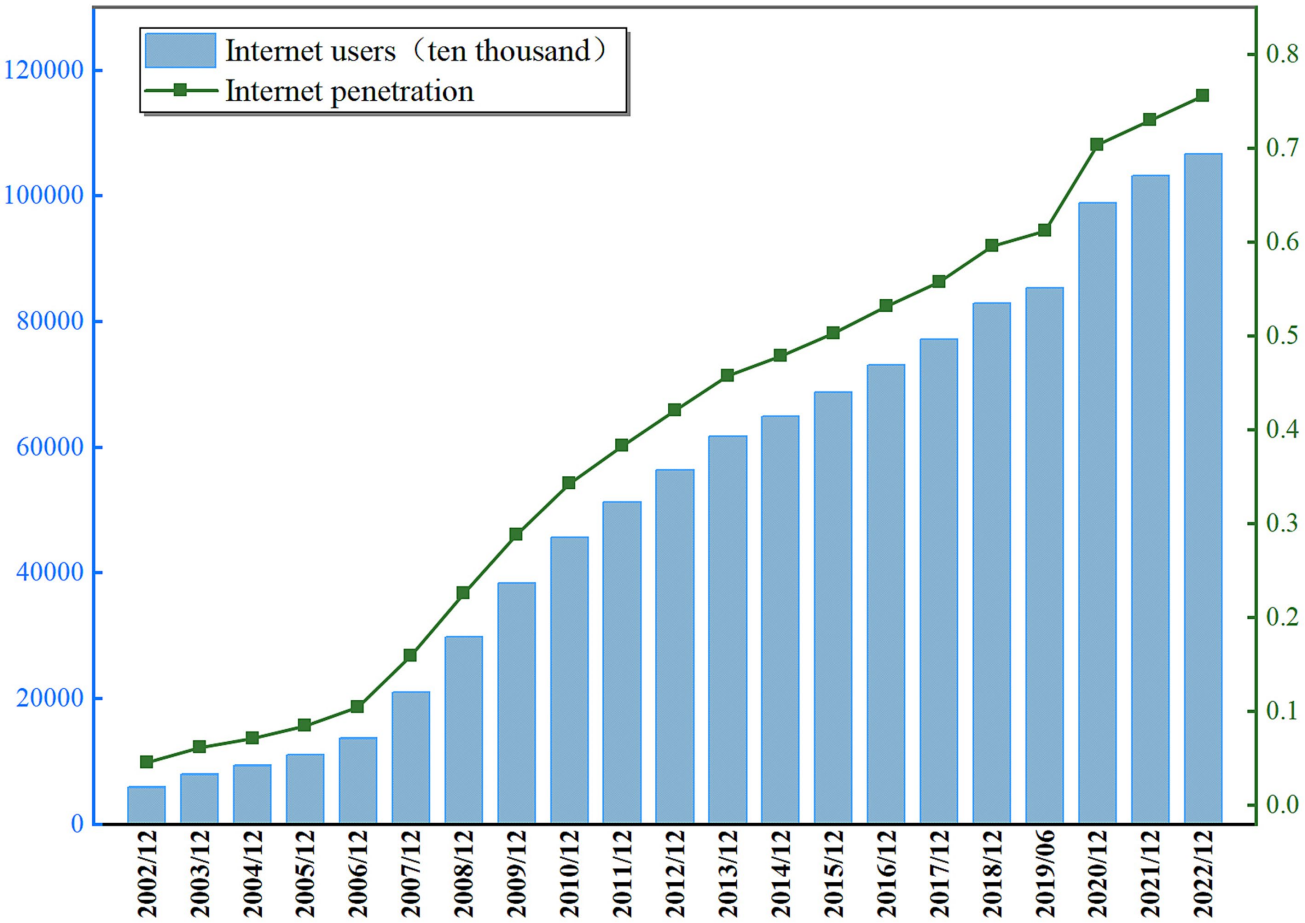
Scale of internet users and internet penetration rate in China, 2002–2022. Data source: China Internet Development Statistical Survey.

Since the 1960s, information technology has developed rapidly and internet applications have accelerated in popularity. This has brought unprecedented and profound changes to human production and lifestyle and even to all areas of the economy and society. As shown in Figure 1, in the past two decades, China’s internet technology has developed rapidly, and as of December 2022, the number of internet users reached 1.067 billion and the internet penetration rate reached 75.6% (1). The popularity and use of the internet have greatly facilitated people’s activities such as shopping, learning, entertainment, socializing and information acquisition (2–5), but at the same time, inappropriate internet use behaviors such as compulsive and problematic internet use and social media distraction have caused a series of psychological problems such as internet addiction and online procrastination (6–12). On this basis, the issue of how to use the internet properly and happily has attracted widespread attention in the academic community.

In recent years, the Chinese government has repeatedly mentioned in its work reports the need to “enhance people’s happiness and sense of access” (13). The issue of “how to enhance the residents’ happiness” has become a hot topic of concern for all sectors of society. So, what is “happiness”? Psychologists subdivide happiness into psychological well-being (PWB) and subjective well-being (SWB) (14), and consider subjective well-being as a comprehensive assessment of people’s satisfaction with life and its various aspects, as well as the resulting psychological state with mainly positive emotions, “internal emotions” and “personal self-evaluation” (15); while psychological well-being refers to a good state of human psychological functioning, including six dimensions which are self-acceptance, positive relationships with others, functional autonomy, environmental control, life goals, and personal growth (16). Based on the hypothesis of rational man, happiness has been equated with maximum utility in traditional economic studies (17), whereas in the current economic context it is considered a manifestation of individual satisfaction (18). In general, although the definition of happiness varies from discipline to discipline, the core content of the existing studies is consistent in terms of research methods and empirical results: happiness is an individual’s overall assessment of his or her quality of life and psychological state according to his or her own criteria, including the so-called happiness (19–21). For a long time, research on the happiness of residents has focused on income, education, social participation, family status and social environment (19, 22–26), and few studies have focused on the impact of the development of information technology on the happiness of residents in developing countries. In recent years, the harmful effects of excessive internet use on the mental health of adolescents and the older adult have been hotly debated (27–31), but this paper discusses the effects of internet use on overall happiness (including quality of life and psychological state) of Chinese residents compared to non-internet use. So, what exactly is the impact of internet use on residents’ happiness? Scholars have discussed this issue extensively and profoundly from various fields, including psychology, economics, and sociology.

On the one hand, existing studies suggest that there is a “network gain effect” that makes internet use increase the level of happiness of residents (5, 32). First, the use of the internet promotes social participation and social capital accumulation, and in the process generates a large number of positive emotions that contribute to residents’ happiness levels. For example, the emergence of Facebook, QQ, and WeChat has increased communication between people who are geographically distant (33), thereby strengthening relationships and social capital. In addition to maintaining and promoting existing interpersonal relationships, SNSs also assist in expanding social networks, which in turn increases social capital accumulation and enhances the happiness of users (34–37). Second, the internet provides more access to information. Pénard et al. (38) argue that the internet enables people to search for information more efficiently, which leads to cheaper goods, more diverse services, and better-matched job opportunities to improve their happiness. Through the internet, people can acquire relevant skills and knowledge to improve their human capital (39). For instance, with the emergence of mobile medical models, people are now able to acquire additional health information through the internet (40, 41). At the same time, the information property of the internet and the information benefits it brings have greatly reduced the cost of information search, enabling workers to obtain more employment information and expand their employment channels at a lower cost (42–45). Internet use has transformed individuals from passive consumers of information to active seekers, resulting in greater control over their lives and a greater sense of happiness (36). In addition, the internet has greatly improved work efficiency and enriched people’s life experience. It allows the possibility of working online, which undoubtedly enhances work efficiency, job satisfaction, and individual happiness (46, 47). Furthermore, the internet facilitates the dissemination of information and the efficient exchange of information between colleagues, which can improve the quality of work (48, 49).

On the other hand, the “network substitution effect” in internet use may lead to social isolation and reduced social competence, as well as information overload, increased anxiety and stress, and thus reduced happiness (38, 50). The use of the internet will lead to an increase in online communication time. This outcome will partly replace face-to-face interaction between individuals, narrowing their social circles and reducing the frequency and quality of communication between family members, thereby leading to disharmonious family relationships and negatively impacting people’s welfare (32, 33, 51–54). A number of researchers have also argued that the use of the internet has significantly altered how individuals perceive and behave in social situations. The perception of an individual’s situation is influenced by their judgment of their own circumstances, their comparisons with others, and their expectations of the future, all of which, in turn, affect their level of happiness (55, 56). When comparing themselves with people who are better off than they are, people experience psychological loss and relative deprivation, which can reduce their sense of happiness. People’s social lives have expanded beyond their everyday lives due to the internet, allowing them to compare their experiences with people from different countries and backgrounds. By doing so, people may change their material desires, change their orientation toward their social class and relative income, and thus negatively affect the level of happiness (33, 57–62). At the same time, excessive use of the internet can also bring a series of negative effects on people’s productive life, affecting people’s mental health, such as social media distractions, online procrastination, etc., which may cause people to work less efficiently in the process of telecommuting, negatively affect productivity, job satisfaction, and general happiness (63, 64).

Although being addicted to the internet can reduce people’s happiness, this study finds that using the internet as a whole will increase people’s happiness compared to not using the internet. It is without doubt that internet use contributes significantly to individual happiness, but its direction and the mechanism by which it does so need to be further investigated empirically. On the basis of theoretical analysis, this study uses data from the 2018 China General Social Survey (CGSS) to empirically test the impact of internet use on the happiness of Chinese residents and its action mechanism. A marginal contribution of this study is that it provides novel empirical evidence as to how internet use affects residents’ happiness. Unlike existing research that takes social capital and information access as intermediate transmission mechanisms, this study examines how social interaction frequency and socioeconomic status (SES) contribute to the impact of internet use on residents’ happiness. By providing novel empirical evidence to further understand the relationship between internet use and residents’ happiness we are able to gain a deeper understanding of this relationship.

The remainder of the paper is organized as follows: Section 2 presents our theoretical analysis and research hypotheses. Section 3 describes the data and methodology used in our research. Section 4 presents the estimation results. Section 5 analyzes the impact mechanisms. Section 6 presents research findings and policy implications.

## 2. Theoretical analysis and research hypotheses

### 2.1. Internet use and resident happiness

Numerous studies have demonstrated that internet use positively impacts the happiness of those who use it (36, 38, 65, 66). By facilitating access to and dissemination of information (38), the internet can enhance the utility level of the population. It will result in increased activities, such as online leisure, entertainment, shopping, and consumption (4, 5), which will in turn improve their welfare. Moreover, the use of the internet is often associated with the consumption of “relationship goods” (67) and an increase in social capital. Relational goods have proven to be a source of personal happiness (53, 68), and an increase in social capital plays the same role (69). The internet likewise helps improve residents’ level of interaction with society as well as enhances their self-evaluation, self-efficacy, and sense of value, which in turn increases their sense of happiness (70–72).

However, it is undeniable that internet use may negatively affect residents’ happiness. For example, it may increase people’s loneliness by reducing the frequency of face-to-face communication and lower welfare; it may also weaken residents’ happiness by increasing personal material aspirations and personal frustration and decreasing their relative income levels (60–62). The results of a comprehensive comparison analysis reveal that internet usage by Chinese residents tends to lead to higher levels of happiness. In light of this finding, we propose the first research hypothesis in this study.

*H1*: The use of the internet significantly improves the happiness of residents.

### 2.2. Role of the frequency of social interaction in the effect of internet use on residents’ happiness

Due to the increasing use of the internet, people’s social interactions have changed rapidly, especially in terms of the frequency and scope of social interactions. The frequency of social interaction deals with the rate at which social contact occurs (73), which is an indicator of the number of times people interact in a certain amount of time. According to related research, higher frequency of social interaction is often thought to be associated with higher happiness. In the era of increasing internet usage, people’s social interactions have undergone rapid changes. Many studies have examined the impact of the internet on residents’ happiness without neglecting the importance of social interactions. As the first study to examine the effects of internet use on social interactions and well-being, Kraut et al. (74) concluded that internet use decreases social interaction with family and friends, thus increasing loneliness and decreasing well-being. Several early studies supported the view that internet usage reduces face-to-face interaction time, which negatively impacts the happiness of individuals (32, 33, 51–54, 75). According to more and more researchers, as ICTs have developed and the internet has become increasingly popular, particularly with the emergence of SNSs, the use of the internet does not entirely replace face-to-face communication with family and friends. By complementing existing social relationships, the internet provides the means of maintaining and developing existing relationships as well as establishing new social connections and making new friends (54, 76, 77). Thus, internet use strengthens and enhances people’s social networks. As one of the manifestations of social capital, the expansion or strengthening of social networks will lead to an increase in social capital, which will in turn enhance residents’ happiness (78, 79). In light of this, we propose the second research hypothesis in this study.

*H2*: Internet use further enhances residents’ happiness by increasing the frequency of their social interactions.

### 2.3. Role of socioeconomic status in the effect of internet use on residents’ happiness

As a platform for the exchange and dissemination of information, the internet has the characteristics of anonymity and equality, which contribute to the transformation of existing social structures and the SES of individuals. SES is a complex and multidimensional concept that reflects the relative position of an individual in a society or group, including both objective measures (e.g., income or education) and subjective assessments of their position (80). SES has been shown to be an influential factor in determining residents’ happiness and the higher the SES, the more resources residents will have at their disposal, resulting in a heightened degree of happiness (81–85).

Information technology, represented by the internet, has greatly influenced the production, lifestyle, and consumption patterns of residents. In some ways, the internet has reduced the cost of searching for information, thereby alleviating information asymmetry. Through it, search-matching costs on the labor market are reduced, residents have more access to information and technical resources, employment channels are expanded, opportunities and rewards on the labor market are enhanced, and their SES is improved. Note that this enhancement effect is pronounced for residents of low SES, which inevitably enhances their happiness (42, 86–88). Furthermore, the emergence of a platform economy built upon the internet has triggered structural changes in the labor market, which has widened the scope of labor employment and entrepreneurship on a global scale. According to Boston Consulting Group’s report, “Year 2035: 400 Million Job Opportunities in the Digital Age,” 415 million jobs will be created worldwide by 2035 in the digital economy sector (89). It is possible for internet technology to drive employment growth through the creation of new tasks, and even to create some new jobs in the process (90). It will also increase employment options for workers, allowing them to earn an adequate income while performing relatively flexible work tasks. This situation will raise their income levels, improve their SES, and ultimately increase their happiness (91). As a result of the above analysis, the third research hypothesis of this study is formulated as follows.

*H3*: Internet use further enhances residents’ happiness by improving their socioeconomic status.

## 3. Variables and data

### 3.1. Variable setting

#### 3.1.1. Dependent variable

The text continues to use data to demonstrate the assumptions put forward earlier. Happiness has been described in many different ways in previous literature (92, 93). It is defined as a sense that includes the absence of negativity, a positive attitude, tranquility, personal development, luck, joy, desires, purpose, and belonging (94). Happiness covers how well individuals are doing in life, including the social, health, material, and subjective dimensions of well-being (95, 96).

As happiness cannot be visually observed and measured, this study uses the question “In general, do you think you are happy in your life?” to indicate the happiness of residents. The scale from 1 to 5 indicates very unhappy, relatively unhappy, unhappy, relatively happy, and very happy, respectively. It also means that as the number increases, the happiness of the residents gradually increases.

#### 3.1.2. Explanatory variable

Internet use can be categorized into academic, social, and recreational (97). At the same time, there are a lot of devices for using the internet. Therefore, in this study, we chose the question “In the past 6 months, have you accessed the internet, including computers, cell phones, smart wear, and other devices?” to indicate internet use. If you use the internet, it is recorded as 1, and if not, it is recorded as 0.

#### 3.1.3. Control variables

Reference is made to previous literature related to the study of the happiness of the population (98–101). To study the effect of internet use on the happiness of the population, we introduced control variables, including gender, age, age squared, ethnicity, religion, literacy, political affiliation, marital status, and household registration status. For gender, males were assigned a value of 1 and females a value of 0. The year of birth of the respondents was asked in the questionnaire, and the year of birth was subtracted from the year of the interview (i.e., 2018) to obtain the age of the respondent. Age was squared by dividing the age by 10 and then squared. For ethnicity, Han was assigned a value of 1 and minority was assigned a value of 0. For religion, having a religion was assigned a value of 1 and not having a religion a value of 0. For education, no education is assigned a value of 1; private schools, literacy classes, and elementary schools are assigned a value of 2; junior high schools are assigned a value of 3; high schools, junior colleges, and technical schools/vocational high schools are assigned a value of 4; university colleges (adult higher education) and university colleges (formal higher education) are assigned a value of 5; and university bachelor’ s degrees and above are assigned a value of 6. In the political status variable, Communist Party (CCP) member is assigned a value of 1 and non-CCP member a value of 0. In the marital status variable, the unmarried and cohabitating are assigned a value of 0; the first married with a spouse, remarried with a spouse, separated and not divorced, divorced, and widowed are assigned a value of 1. In the household status variable, residents who have agricultural households are assigned a value of 0; otherwise, they are assigned a value of 1.

### 3.2. Description of data

The data used in this paper come from the Chinese General Social Survey (CGSS) released by the China Survey and Data Center of Renmin University of China. Started in 2003, the CGSS adopted a multi-order stratified unequal probability sampling method to survey individuals in 125 counties (districts), 500 streets (townships), 1,000 neighborhood (village) committees, and 10,000 households nationwide. In this study, the latest published CGSS data of 2018 were selected, forming a total of 12,787 valid samples. We normalized the raw data. On the one hand, the data in this paper is processed with positive criteria. For example, in the original questionnaire, “In the last 6 months, have you been on the internet, including using various devices such as computers, cell phones and smart wears?”, which marks the answer “yes” as 1, and the answer “no” as 2. We replaced 2 with 0. In addition, happiness, gender, age, ethnicity, religion, education, political status, marital status, household status, frequency of social interactions and socioeconomic status are all positive indicators. On the other hand, we use the mvdecode command to handle the special values in the questionnaire responses. According to Table 1, the correlation coefficient between internet use and residents’ happiness reached 0.062, and the *p*-value between internet use and residents’ happiness is 0, indicating a strong correlation between internet use and residents’ happiness. This implies that the use of the internet will increase the happiness of the residents, verifying the basic hypothesis of this paper.

**Table 1:** Matrix of correlations.

Variables	(1)	(2)
(1) Internet use	1.000	
(2) Happiness	0.062^***^	1.000

*, **, and *** indicate significance at the 10%, 5%, and 1% confidence levels, respectively, and the values of the statistics are in parentheses.

The statistical description of the variables is shown in Table 2. The happiness of the residents is measured between saying that they are not happy or unhappy and relatively happy; the percentage of residents who use the internet is 61.9%. In terms of demographic characteristics, the proportion of males was 46.7%, the average age was 52 years old, the proportion of Han Chinese was 92.7%, the proportion of those with religious beliefs was 10.7%, the education level was between college specialist and college or above; the proportion of political appearance was 11.1% for CCP members; the proportion of married was 87.8%; and the proportion of residents that do not have agricultural households was 45.3%.

**Table 2:** Statistical description of the variables.

Variables	Mean	Median	SD	Min	Max	*n*
Happiness	3.897	4.000	0.816	1.000	5.000	12751.000
Internet use	0.619	1.000	0.486	0.000	1.000	12745.000
Gender	0.467	0.000	0.499	0.000	1.000	12764.000
Age	51.855	52.000	16.936	18.000	118.000	12764.000
Age squared	29.757	27.040	17.745	3.240	139.240	12764.000
Nationality	0.927	1.000	0.259	0.000	1.000	12764.000
Religious belief	0.107	0.000	0.309	0.000	1.000	12764.000
Education	3.155	3.000	1.502	1.000	6.000	12735.000
Political status	0.111	0.000	0.314	0.000	1.000	12764.000
Marital status	0.879	1.000	0.327	0.000	1.000	12764.000
Household status	0.453	0.000	0.498	0.000	1.000	12764.000

## 4. Empirical analyses

### 4.1. Baseline regression results

This study first empirically tests the effect of internet use on residents’ happiness using a least squares regression model. In addition, because the explanatory variables are ordered discrete variables, ordered logit and ordered probit models are used to regress the cross-sectional data on ordered discrete variables and test the robustness of the results. The corresponding regression equations are set as follows.


(1) $$\mathrm{Happiness}=\beta +\beta_{1}*\mathrm{Internet}\;\mathrm{use}+\beta_{2}*\mathrm{Control}+\varepsilon \;$$

*Happiness* denotes residents’ happiness; *internet use* denotes the internet use; *Control* denotes the control variables, including individual demographic characteristics variables (i.e., gender, age, square of age, nationality, religion, education, political status, marital status, and household status); and *ε* is the random error term.

Table 3 reports the corresponding regression results. Column (1) represents the results of the regression model using the ordinary least squares (OLS) method, which does not control for demographic characteristics of the population, showing that the positive effect of internet use on the happiness of the population is significant at the 1% level, but the adjusted *R*-squared is only 0.004. After controlling for the demographic characteristics of the population, as shown in column (2) of the table, the results are similar to those in column (1), with a higher value of adjusted *R*-squared. The coefficients of the happiness variable are also significantly positive in both the Ologit and Oprobit regressions, indicating that internet use significantly increases the happiness of the population.

**Table 3:** Baseline regression results of the effect of internet use on the happiness of Chinese residents.

	Ols (1)	Ols (2)	Ologit (3)	Ologit (4)	Oprobit (5)	Oprobit (6)
Internet use	0.105^***^ (7.05)	0.119^***^ (5.87)	0.184^***^ (5.08)	0.263^***^ (5.23)	0.115^***^ (5.68)	0.148^***^ (5.30)
Gender		−0.0531^***^ (−3.62)		−0.148^***^ (−4.11)		−0.0804^***^ (−3.97)
Age		−0.0241^***^ (−8.74)		−0.0586^***^ (−8.60)		−0.0326^***^ (−8.54)
Age squared		0.0265^***^ (10.52)		0.0653^***^ (10.44)		0.0362^***^ (10.37)
Nationality		−0.0159 (−0.56)		−0.0505 (−0.72)		−0.0250 (−0.64)
Religious belief		0.0583^**^ (2.45)		0.178^***^ (3.02)		0.0944^***^ (2.88)
Education		0.0454^***^ (6.67)		0.0991^***^ (5.91)		0.0570^***^ (6.05)
Political status		0.123^***^ (4.99)		0.331^***^ (5.49)		0.192^***^ (5.58)
Marital status		0.198^***^ (7.34)		0.496^***^ (7.56)		0.265^***^ (7.17)
Household status		0.0453^***^ (2.65)		0.121^***^ (2.87)		0.0682^***^ (2.89)
Constant	3.833^***^ (327.62)	3.969^***^ (52.04)				
Adj *R*^2^	0.004	0.033				
Pseudo *R*^2^			0.001	0.015	0.001	0.015
*n*	12,732	12,703	12,732	12,703	12,732	12,703

^*^, ^**^, and ^***^ indicate significance at the 10%, 5%, and 1% confidence levels, respectively, and the values of the statistics are in parentheses (the next table is the same as).

The regression results for other control variables are generally consistent with the existing literature as well. First, the coefficient of gender is negative and significant at the 1% level, indicating that women’s happiness is higher than that of men. In China, men can enjoy better education, higher employment rates and income. This results in higher social expectations for men. So men will face more pressure, which may reduce their happiness (102). For women, although they are more prone to negative emotions due to their physiology, it does not mean that they are unhappy (15). Moreover, society empowers women more, and higher income levels enhance their status in the family and give them more freedom (103). Second, the coefficient of age is positive and significant at the 1% level, indicating that the happiness of residents gradually decreases with age. The squared coefficient of age is significantly positive, indicating that age and happiness have a U-shaped relationship (104). Happiness decreases from a high point at a young age, reaches a low point at middle age, increases thereafter, and reaches another high point at old age. Third, the coefficient of ethnicity is not significant, indicating that the presence of ethnic minorities does not have an effect on the happiness of residents. Fourth, the coefficient of religiosity is significantly positive, indicating that residents with religious beliefs have a stronger sense of happiness. Religious beliefs have the function of guiding the thoughts and behaviors of individuals, and the spiritual protection function of religious culture is conducive to making human beings perceive happiness (105). Fifth, the coefficient of education is positive and significant at the 1% level, which indicates that the increase of education level will significantly improve the happiness of residents. Education not only enriches people’s spiritual world and positively affects their self-esteem, self-confidence, and happiness, but also enhances their happiness by increasing their income level (106). Sixth, the coefficient of political affiliation is positive and significant at the 1% level, indicating that residents who are members of the CCP are happier. Being a CCP member is considered to be important social capital (107). Studies show that CCP and the Communist Youth League of China (CYLC) members are less money conscious than the masses and will be more self-conscious and participate more in social activities (108). Seventh, the coefficient of marital status is positive and significant at the 1% level, indicating that married residents are happier. After several years of follow-up and research development, marriage makes people happier. On the one hand, due to the national welfare policy, married couples can enjoy tax incentives (109); on the other hand, marriage alliances can be more rewarding (110). Eighth, a significantly positive household coefficient indicates that the happiness of non-rural household residents is stronger. Residents of urban households have better quality social services and health care coverage, while rural residents suffer from greater life stress, and overall, the happiness of rural household residents is significantly lower than that of non-rural households (111).

### 4.2. Robustness test

Internet usage frequency is a good indicator of internet usage. Therefore, this study selected internet usage frequency as a proxy variable for the explanatory variable of internet usage. The question in the questionnaire asks, “In the past year, how often did you use the internet (including cell phone access)?” The answers from 1–5 indicate never, rarely, sometimes, often, and very often, respectively, which indicate the internet use of residents. Table 4 reports the regression results. The regression coefficients of happiness in the least squares regression model are all positive and significant at the 1% level. Similarly, the coefficients of happiness variables in the Ologit and Oprobit regressions are all significantly positive, indicating that the increase in the frequency of internet use significantly enhances the happiness of residents.

**Table 4:** Effect of frequency of internet use on residents’ happiness.

Explanatory variables	Ols (1)	Ols (2)	Ologit (3)	Ologit (4)	Oprobit (5)	Oprobit (6)
Internet usage frequency	0.0320^***^ (7.46)	0.0335^***^ (5.38)	0.0575^***^ (5.49)	0.0752^***^ (4.88)	0.0357^***^ (6.11)	0.0423^***^ (4.92)
Gender		−0.0546^***^ (−3.73)		−0.151^***^ (−4.20)		−0.0821^***^ (−4.06)
Age		−0.0224^***^ (−8.06)		−0.0547^***^ (−7.95)		−0.0305^***^ (−7.91)
Age squared		0.0248^***^ (9.90)		0.0614^***^ (9.88)		0.0340^***^ (9.81)
Nationality		−0.0172 (−0.61)		−0.0530 (−0.76)		−0.0264 (−0.68)
Religious belief		0.0586^**^ (2.47)		0.180^***^ (3.04)		0.0946^***^ (2.89)
Education		0.0433^***^ (6.26)		0.0939^***^ (5.51)		0.0541^***^ (5.65)
Political status		0.126^***^ (5.12)		0.338^***^ (5.60)		0.196^***^ (5.70)
Marital status		0.200^***^ (7.42)		0.498^***^ (7.59)		0.267^***^ (7.24)
Household status		0.0490^***^ (2.87)		0.130^***^ (3.07)		0.0726^***^ (3.08)
Constant	3.805^***^ (264.88)	3.914^***^ (49.31)				
Adj *R*^2^	0.004	0.032				
Pseudo *R*^2^			0.001	0.015	0.001	0.015
*n*	12,745	12,716	12,745	12,716	12,745	12,716

*, **, and ***indicate significance at the 10%, 5%, and 1% confidence levels, respectively, and the values of the statistics are in parentheses.

### 4.3. Endogenous issues

The estimated results may have endogeneity problems due to reverse causality and omitted variables. The endogeneity problem cannot be ignored because residents have more leisure time to use the internet when their happiness is stronger. In this study, we use an instrumental variable regression model to deal with the endogeneity problem; the selected instrumental variable is whether or not to own a cell phone independently. The question in the questionnaire is, “Do you have a cell phone that you use alone?” Since having a cell phone alone or not does not directly affect their happiness, but having a cell phone alone is associated with the internet use of residents, this instrumental variable is theoretically feasible.

Table 5 reports the results of the two-stage instrumental variable estimates of internet use on happiness. In the 2SLS regression results, the non-identifiable test statistic is 378.590, which rejects the original hypothesis that the instrumental variable is not correlated with the endogenous variable at the 1% significance level. The weak instrumental variable statistic is 389.889, which is greater than the critical value at the 10% bias level of 16.38 (112). Therefore, the original hypothesis of the existence of a weak instrumental variable is rejected; the *t*-value of the instrumental variable is 19.57, indicating that the instrumental variable is valid. From the first-stage regression results, the coefficient of independent cell phone ownership is significantly positive, indicating that independent cell phone ownership promotes internet use. The results of the second-stage regression indicate that internet use significantly enhances residents’ happiness after controlling for endogeneity issues, so the results are robust. To further verify the rationality of the instrumental variable selection, the Oprobit instrumental variable model is used to test the robustness of the two-stage regression results, where atanhrho_12 is significant at the 1% level of significance, thus rejecting the original hypothesis that internet use is an exogenous variable. The Wald test statistic is 13260.28, which is significant at the 1% level, indicating that the instrumental variable has explanatory power. The results of the first-stage regression indicate that independent ownership of a cell phone significantly promotes internet use, and the results of the second-stage regression show the same significant positive effect of internet use on residents’ happiness after controlling for endogeneity issues.

**Table 5:** Internet use and happiness: two-stage instrumental variable estimates.

Explanatory variables	2SLS		IVoprobit	
	Internet use	Happiness	Internet use	Happiness
	(1)	(2)	(3)	(4)
Internet use		0.513^***^ (4.300)		0.641^***^ (4.210)
Ownership of cell phones	0.228^***^ (19.570)		0.229^***^ (19.830)	
Control variables	Yes	Yes	Yes	Yes
Unrecognizable test	378.590			
Weak instrumental variable test	389.889			
Wald test			13260.280	
atanhrho_12			−0.178^***^ (−3.210)	
Sample size	12,695	12,695	12,716	12,716

*, **, and *** indicate significance at the 10%, 5%, and 1% confidence levels, respectively, and the values of the statistics are in parentheses.

## 5. Analysis of intermediary effect mode

### 5.1. Model and variables

The results in the previous section show that internet use has a significant positive effect on residents’ happiness. So how does internet use enhance residents’ happiness? Based on the principle of the mediating effect test proposed by Baron and Kenny (113), the present study constructs the following mediating effect equation to empirically test the mechanism of the effect of internet use on residents’ happiness:


(2) $$\mathrm{Happiness}=\delta +a_{1}*\mathrm{Internet}\;\mathrm{use}+b*\mathrm{Control}+\varepsilon$$

(3) Mediator=δ+c∗Internetuse+b∗Control+ε

(4) $$\begin{array}{l} \mathrm{Happiness}=\delta +a_{2}*\mathrm{Internet}\;\mathrm{use}+e*\mathrm{Mediator}+\\ b*\mathrm{Control}+\varepsilon \end{array}$$

Eq. (2) tests the effect of internet use on residents’ happiness which is the same as the baseline regression Eq. (1). The second step regresses model (3), and if the regression coefficient *c* is significant, it indicates that internet use has a significant effect on the mediating variable and then the third step regresses model (4). *a*_1_ is the total effect of internet use on happiness, and *a*_2_ is the direct effect of internet use on happiness. If the coefficients *c* and *e* are significant, then it means there is a partial mediating effect of the mediating variable; if the coefficients *c* and *e* are significant but the coefficient *a*_2_ is not significant, then it indicates that there is a full mediating effect of the mediating variable. In addition, this study further uses the Sobel method to test the existence and magnitude of the mediating effect. Since the explanatory variables in Eqs. (2)–(4) are all discrete variables, to enhance the reliability of the results, Ologit and Oprobit models are used, respectively, in this study for regression. The study likewise uses the Sobel method to further test the existence and magnitude of mediating effects.

This study analyzes the mechanism of social interaction frequency and SES. The frequency of social interaction is based on the questionnaire, “In a typical day, how many people, other than family members and relatives, do you have contact with in total through various means? Contact refers to one-to-one contact, including meeting, telephone, e-mail, WeChat, etc., whether you know them or not.” The responses from 1–7 indicate 0, 1–4, 5–9, 10–19, 20–49, 50–99, and 100 or more times, respectively. For SES, this study uses the household economic status in the questionnaire to express “What is the economic status of your household in your region?” The responses from 1 to 5 indicate far below average, below average, average, above average, and far above average to measure the household economic status, respectively.

The statistical descriptions of the mediating variables are shown in Table 6. On average, the frequency of social interactions ranged from 5 to 19; SES was between below average and average.

**Table 6:** Statistical description of the mediating variables.

Variables	Mean	Median	SD	Min	Max	*n*
Frequency of social interactions	3.049	3.000	1.383	1.000	7.000	4431.000
socioeconomic status	2.577	3.000	0.729	1.000	5.000	12638.000

### 5.2. Empirical results

The results of the mediating effects of social interaction frequency are shown in Table 7. The regression results of the Ologit and Oprobit models are consistent, indicating that the results are robust. In the first step, the baseline regression results indicate that the total effect of internet use significantly enhances residents’ happiness. In the second step, the coefficients of internet use are also all significantly positive, indicating that internet use increases the frequency of social interactions among residents and enhances SES. In the third step, the coefficients before social interaction frequency and SES are both significantly positive, indicating that the increase in social interaction frequency and SES significantly improves residents’ happiness. Meanwhile, the coefficients before internet use are significantly positive, implying that social interaction frequency and SES play a role of partial mediating effect between the effect of internet use on residents’ happiness. There is a transmission mechanism in the process of internet use affecting residents’ happiness which is internet use → social interaction frequency/SES → happiness. To further ensure the robustness of the regression results, this study uses the Sobel method for testing. The absolute value of Sobel *Z* size is 2.890 and 9.922, which are significant at the 1% significance level, where the mediating effect accounts for 0.064 and 0.445 of the total effect, indicating that there is a significant mediating effect of social interaction frequency and SES. Hence, the theoretical hypotheses 2 and 3 were verified.

**Table 7:** Mediating effects of frequency of social interactions and socioeconomic status.

Variables	Ologit			Oprobit		
	Step 1	Step 2	Step 3	Step 1	Step 2	Step 3
	Happiness (1)	Inter-mediate variables (2)	Happiness (3)	Happiness (4)	Inter-mediate variables (5)	Happiness (6)
Internet use	0.263^***^ (5.23)	0.429^***^ (5.48)	0.284^***^ (3.31)	0.148^***^ (5.30)	0.256^***^ (5.71)	0.165^***^ (3.46)
Frequency of social interactions			0.076^***^ (3.35)			0.042^***^ (3.32)
Control variables	Yes	Yes	Yes	Yes	Yes	Yes
Pseudo *R*^2^	0.015	0.034	0.017	0.015	0.035	0.017
*n*	12,703	4,419	4,416	12,703	4,419	4,416
Internet use	0.263^***^ (5.23)	0.502^***^ (10.22)	0.141^***^ (2.77)	0.148^***^ (5.30)	0.291^***^ (10.38)	0.0821^***^ (2.89)
Socioeconomic status			0.690^***^ (25.99)			0.387^***^ (26.62)
Control variables	Yes	Yes	Yes	Yes	Yes	Yes
Pseudo *R*^2^	0.015	0.043	0.039	0.015	0.044	0.040
*n*	12,703	12,591	12,579	12,703	12,591	12,579

*, **, and *** indicate significance at the 10%, 5%, and 1% confidence levels, respectively, and the values of the statistics are in parentheses.

## 6. Research findings and policy implications

This study investigates the impact of internet use on residents’ happiness based on data from the 2018 CGSS. The empirical results show that, first, internet use significantly enhances residents’ happiness. A two-stage instrumental variable estimation is conducted using whether or not a person independently owns a cell phone as an instrumental variable, and the finding still holds after overcoming the endogeneity problem and conducting a series of robustness tests. Second, the mediating effect model test finds that there is a transmission mechanism in the process of internet use affecting residents’ happiness which is internet use → social interaction frequency/SES → happiness. Internet use significantly increases the social interaction frequency of residents and enhances their SES, and the increase in social interaction frequency and SES significantly enhances residents’ happiness.

The following recommendations are proposed based on the conclusions of this article:

Firstly, spread internet use more widely while giving individuals more internet literacy. As of June 2022, China’s internet penetration rate was only 74.4%, which is still a large gap compared to developed countries such as Europe and the United States. Therefore, it is necessary to further increase the internet penetration rate and improve people’s internet usage skills by improving internet infrastructure to solve the problem of internet usage difficulties for the poor and the old, lower tariffs, accelerate the cultivation of internet-related talents and popularise internet education, enabling more people to benefit from the convenience resulting from internet development and making internet usage an effective means of enhancing residents’ happiness.

Secondly, the interactive effect of the internet should be brought into full play to increase the frequency of social interaction among residents. Internet popularization should differ depending on the age group of residents. For young people, digital teaching and high-quality digital resources should be developed, providing strong support for promoting the reform and development of basic education in the information era. For the older adult, the age-appropriate transformation of applications such as WeChat, Tiktok, Alipay, and so on can expand their family network and friend network, and reduce their sense of loneliness. Meanwhile, the disabled and other special groups could cross the “digital divide” and improve their sense of happiness with the help of the accessibility transformation of applications. In addition, “internet + tourism,” “internet + medical,” “internet + education” and other “internet +” platforms should be vigorously developed to provide people with convenience and enrich their spiritual and cultural lives so that people could feel happier as technology advances. Moreover, the internet, as a social media, plays a significant role in the development and maintenance of social relationships. As such, it is important to encourage individuals to engage in more social activities via the internet, expand their social networks, and strengthen their social capital, while also promoting pro-social behaviors.

Thirdly, it is important to develop individuals’ understandings of the internet, to guide them in using the internet rationally, to create an upwardly mobile online environment, and to improve individual socioeconomic status. First and foremost, it is necessary to diversify the types and disciplines of online education, develop digital labor platforms, explore the potential of the internet to enhance work efficiency and flexibility. Assist people in using the internet rationally and scientifically in their studies, work, and daily lives to improve their skills and knowledge, to avoid playing online games or excessive use of virtual social networks, and to increase awareness of the possibility of promoting one’s human capital quality through the internet, and to lay a solid foundation for social and economic improvement. Secondly, the relevant departments should grasp the importance of strict supervision, network platforms should consolidate the main responsibilities, and most internet users should improve their network literacy. It would allow people to reduce information asymmetry, broaden employment channels, and increase the income of urban and rural residents through the internet, resulting in an olive-shaped structure of income and wealth distribution with a large middle and two small ends. Expand the middle-income group and enhance the degree of common prosperity so that the internet can truly be used to improve the quality of life of people.

## Data availability statement

Publicly available datasets were analyzed in this study. This data can be found at: http://www.cnsda.org/index.php?r=projects/view&id=35694191.

## Author contributions

YS and XZ: conceptualization, validation, formal analysis, writing—review and editing, and supervision. YS, JG, XZ, and YC: methodology. JG and YC: software. YS, JG, and YC: data curation. YS and JG: writing—original draft preparation. YC: visualization. YS: project administration and funding acquisition. All authors contributed to the article and approved the submitted version.

## Funding

This research was supported by the National Social Science Fund of China (18BTJ019).

## Conflict of interest

The authors declare that the research was conducted in the absence of any commercial or financial relationships that could be construed as a potential conflict of interest.

## Publisher’s note

All claims expressed in this article are solely those of the authors and do not necessarily represent those of their affiliated organizations, or those of the publisher, the editors and the reviewers. Any product that may be evaluated in this article, or claim that may be made by its manufacturer, is not guaranteed or endorsed by the publisher.
